# Janus kinase inhibitors in systemic lupus erythematosus: implications for tyrosine kinase 2 inhibition

**DOI:** 10.3389/fmed.2023.1217147

**Published:** 2023-06-29

**Authors:** Dionysis Nikolopoulos, Ioannis Parodis

**Affiliations:** ^1^Division of Rheumatology, Department of Medicine Solna, Karolinska Institutet, Stockholm, Sweden; ^2^Medical Unit of Gastroenterology, Dermatology, and Rheumatology, Karolinska University Hospital, Stockholm, Sweden; ^3^Department of Rheumatology, Faculty of Medicine and Health, Örebro University, Örebro, Sweden

**Keywords:** systemic lupus erythematosus, JAK inhibitors, TYK2, treatment, small molecules

## Abstract

Aberrant activation of the Janus kinase (JAK) and signal transducer and activator of transcription (STAT) pathway is common in systemic lupus erythematosus (SLE), conferring immune-mediated properties in target tissues. Multiple cytokines activate different combinations of JAKs and STATs to alter the cell fate of target tissue and induce end-organ damage. Thus, the simultaneous blockade of several different cytokines by small molecules acting downstream intracellular signalling has gained traction. JAK inhibitors have been approved for the treatment of several rheumatic diseases, yet hitherto not for SLE. Nevertheless, JAK inhibitors including tofacitinib, baricitinib, and deucravacitinib have shown merit as treatments for SLE. Tofacitinib, a JAK1/3 inhibitor, reduced cholesterol levels, improved vascular function, and decreased the type I interferon signature in SLE patients. Baricitinib, a JAK1/2 inhibitor, demonstrated significant improvements in lupus rashes and arthritis in a phase 2 and a phase 3 randomised controlled trial, but the results were not replicated in another phase 3 trial. Deucravacitinib, a selective tyrosine kinase 2 (TYK2) inhibitor, yielded greater response rates than placebo in a phase 2 trial of SLE and will be investigated in larger phase 3 trials. TYK2 is activated in response to cytokines actively involved in lupus pathogenesis; this review highlights the potential of targeting TYK2 as a promising therapy for SLE.

## 1. Introduction

Systemic lupus erythematosus (SLE) is a chronic autoimmune disease that affects multiple organs, leading to detrimental effects on patients’ quality of life and survival (1). Although its pathogenesis is complex and not yet fully understood, multiple cytokines are believed to underlie pathogenetic pathways. Due to the multifactorial aetiology and heterogeneity of the disease, numerous drugs have been tested in clinical trials with inadequate efficacy (2).

Over the last few decades, several biologic therapies have been developed to target specific inflammatory molecules and pathways. These drugs have shown encouraging results in rheumatic diseases, particularly rheumatoid arthritis (RA), psoriatic arthritis, and ankylosing spondylitis (3). However, many biologics fail to induce short- and long-term remission in rheumatic diseases, which combined with the potential adverse effects underscores the need for new therapies (4). In recent years, small molecules blocking intracellular downstream signalling molecules have shown capacity to change the natural history of several diseases (5).

**Figure 1:**
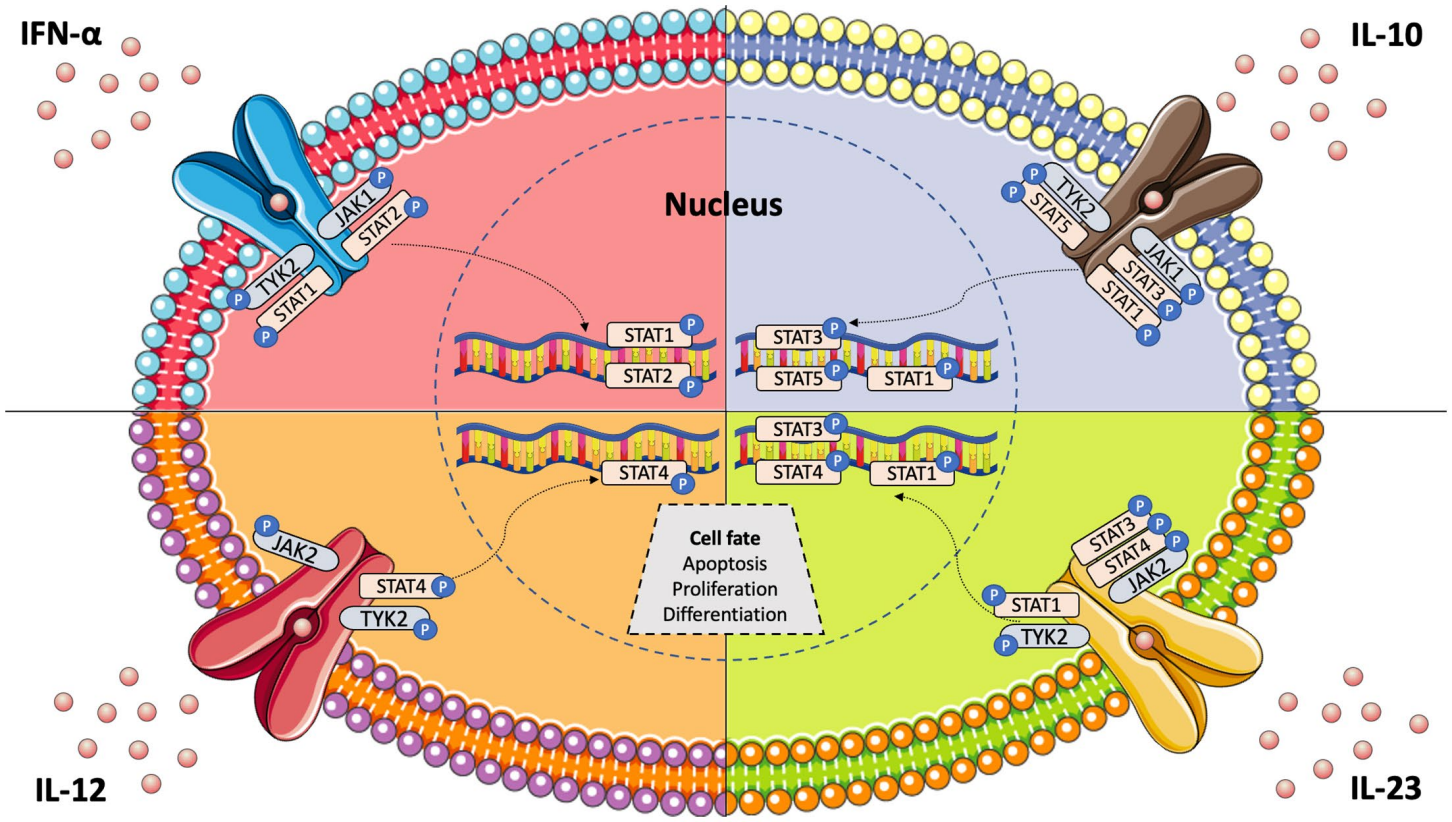
JAK–STAT pathway signalling and key cytokines involved in systemic lupus erythematosus pathogenesis.

Janus kinases (JAKs) are intracellular tyrosine kinases that mediate cytokine-mediated signalling *via* the phosphorylation of other proteins, particularly signal transducer and activator of transcription (STAT) proteins (6). There exist four JAK family members, i.e., JAK1, JAK2, JAK3, and tyrosine kinase 2 (TYK2), and seven different types of STAT proteins, i.e., STAT1, STAT2, STAT3, STAT4, STAT5a, STAT5b, and STAT6. Upon the binding of a ligand, e.g., a cytokine, to a receptor, JAKs activate STAT proteins through phosphorylation (dimers), allowing them to translocate to the nucleus and induce transcription of target genes; this describes the so-called JAK–STAT pathway (7) (Figure 1). The STAT dimers bind promoters of specific genes that control biological processes such as differentiation, proliferation, and apoptosis (6). Notably, each cytokine may activate different JAKs, while each JAK can interact with various STAT proteins, as shown in Table 1.

**Table 1:** Pro-inflammatory cytokines involved in systemic lupus erythematosus pathogenesis and associated Janus kinase (JAK) and signal transduction and activator of transcription (STAT) proteins.

Cytokines	JAKs	STATs
IFN-α	JAK1, TYK2	STAT1, STAT2
IFN-γ	JAK1, JAK2	STAT1
IL-2	JAK1, JAK3	STAT5
IL-6	JAK1, JAK2, TYK2	STAT1, STAT3
IL-10	JAK1, TYK2	STAT1, STAT3, STAT5
IL-12	JAK2, TYK2	STAT4
IL-19	JAK1, JAK2	STAT1, STAT3
IL-20	JAK1, JAK2	STAT1, STAT3
IL-22	JAK1, TYK2	STAT1, STAT3, STAT5
IL-23	JAK2, TYK2	STAT1, STAT3, STAT4

The JAK–STAT pathway is involved in the control of various biological processes, such as haematopoiesis, stem cell fate, and inflammatory response (6). In autoimmune diseases, multiple circulating cytokines activate different combinations of JAKs/STATs to alter the cell fate of target tissues and induce end-organ damage (3). We herein overview the involvement of the JAK–STAT pathway in the pathogenesis of SLE and discuss therapeutic options targeting this pathway, with an emphasis on TYK2 inhibition.

## 2. Rationale for targeting Janus kinases in SLE

SLE is characterised by a plethora of circulating pro-inflammatory cytokines including interferon (IFN)-α, IFN-γ, interleukin (IL)-2, IL-6, IL-10, IL-12/IL-23, IL-19, IL-20, and IL-22 (8). These cytokines orchestrate autoimmunity by pleiotropic mechanisms, particularly through differentiation of immune cells to an autoreactive stage, e.g., IL-6 promoting Th17 formation (9), antibody production, e.g., IFN-α exaggerating formation of plasmablasts (10), and rapid proliferation of autoreactive cells. Although each one of these cytokines exerts several unique inflammatory functions, they use common JAKs and STAT proteins to transduce intracellular signals (3). Thus, the simultaneous blockade of many different cytokines by small molecules acting downstream on intracellular signalling gained traction a few years ago, and JAK inhibitors are now approved for the treatment of several rheumatic diseases, yet hitherto not for SLE (11).

To date, two targeted biologic therapies have been approved for the treatment of SLE, i.e., belimumab and anifrolumab (12). However, many biologics failed to reach the primary outcomes in a multitude of clinical trials, partly owing to the multiple and different immune-mediated pathways that are involved in lupus pathogenesis. Hence, the JAK–STAT pathway provides a rational target since it is activated by several cytokines in underlying aetiology, particularly interferons.

IFN-α is a key cytokine in SLE and other immune-mediated diseases, and its signalling is mediated by the intracellular kinases JAK1 and TYK2 (13) (Figure 1). Tofacitinib, a JAK1/JAK3 inhibitor, yielded some improvement in SLE patients in a phase 1 clinical trial (14). However, baricitinib, a JAK1/JAK2 inhibitor, failed to improve SLE disease activity in one of two randomised phase 3 trials (15, 16). To this end, a selective TYK2 inhibitor (deucravacitinib) has been developed and is being tested for its efficacy in autoimmune diseases, including SLE (17). Deucravacitinib selectively targets TYK2, which is activated in response to IFN-α, IL-6, IL-10, and IL-12/23. Importantly, all these cytokines are actively involved in lupus pathogenesis. Thus, TYK2 inhibition theoretically represents an intriguing therapeutic option for SLE.

## 3. Janus kinase–STAT pathway in SLE

### 3.1. Experimental models

#### 3.1.1. Janus kinase 1–3

New Zealand black/New Zealand white F1 (NZB/W-F1) and Murphy Roths Large/lymphoproliferation (MRL/lpr) murine models of SLE are commonly used to study pathogenetic aspects of lupus nephritis (LN). Experimental studies have shown that IFNs induce activation of STAT1 in glomerular mesangial cells (18), which is consistent with the increased expression of STAT1 seen in glomerular and tubular cells of LN kidney biopsies (19). The administration of a JAK2-STAT1 inhibitor in MRL/lpr mice has been shown to substantially improve lupus disease activity with respect to nephritis, vasculitis, and sialadenitis. Specifically, JAK2-STAT1 inhibition led to decreased levels of cytokines, chemokines, and IgG/C3 depositions in glomerular cells, along with improvements in serological markers of renal function (20). Early treatment with this inhibitor could prevent the establishment of LN in the same murine model, while the administration of JAK2-STAT1 inhibitors in NZB/W-F1 mice with established renal disease increased overall survival, decreased proteinuria, and improved histological renal features (21). Mechanistically, decreased expression of pSTAT3 and IFN-related genes (Mx1, Stat1, Isg15, and Ifit1) along with a profound reduction in the proportion of long-lived plasma cells in both spleen and bone marrow may account for the therapeutic efficacy of JAK2 inhibitors (22, 23).

Another experimental study tested the effectiveness of tofacitinib in the NZB/W-F1 model with regard to renal disease. Tofacitinib-treated mice showed renal histological improvements, decreased C3 and IgG glomerular deposits, significant reductions in the proportion of macrophages and T cells, and a decreased production of pro-inflammatory cytokines. The efficacy of tofacitinib was comparable to that of cyclophosphamide and mycophenolate mofetil (24). The potential therapeutic effect of tofacitinib in LN was validated in the MRL/lpr model of lupus (25). Another inhibitor targeting JAK1 and STAT3 showed ability to prevent LN development when given prophylactically and improve disease activity in established LN (26).

STAT3 is believed to play a pivotal role in LN pathogenesis. Indeed, STAT3 knock-out lupus-prone mice develop milder renal disease with regard to immune component infiltration and deposits in the kidneys (27). In addition, selective STAT3 inhibition has been shown to ameliorate LN both in terms of glomerulonephritis and tubulointerstitial inflammation in murine models of lupus (28). Although several experimental studies suggest that the JAK–STAT pathway represents a promising target in LN, clinical studies are required to confirm this hypothesis.

IFN signalling is an important mediator of cutaneous lupus (29). Ruxolitinib, a relatively specific inhibitor of JAK1 and JAK2, was administered to the MLR/lpr model to investigate its effect on lupus-related skin lesions. This study showed that ruxolitinib ameliorated cutaneous lupus by reducing the expression of IFN-related genes, immune cell infiltration (particularly T cells), and epidermal hyperplasia. However, no favourable effects were reported in other target organs (19). Ruxolitinib has also been successfully used to treat a patient with chilblain lupus (30).

Inhibition of the JAK–STAT pathway is associated with a decrease in autoantibody titres. More specifically, lupus-prone mice treated with JAK2-STAT1 inhibitors, JAK1-STAT3 inhibitors, selective STAT3 inhibitors, and tofacitinib showed reduced serum levels of anti-dsDNA antibodies (20, 24, 27). Across different murine models of lupus treated with JAK/STAT inhibitors, other antibodies such as antinuclear antibodies, anti-Smith, and anti-snRNP were also found to decrease (23, 26). Moreover, STAT3 and JAK2 inhibitors have been shown to increase serum C3 levels in the MRL/lpr model (22).

#### 3.1.2. Tyrosine kinase 2

The TYK2 SNP rs34536443 (TYK2^P^) has been shown to confer a protective effect against autoimmunity (31). Humans expressing this SNP exhibit decreased switched memory B cells, T follicular helper cells, and IFN-mediated signalling. The underlying mechanism has been investigated in knock-out murine models for this allele (32). Homozygous mice for TYK2^P^ have been shown to exhibit decreased T cell IL-12/IL-23-mediated signalling and Th1 and Th17 differentiation. These mice produce decreased proportions of IL-17+/IFN-γ + CD4+ T cells in draining lymph nodes. Taken together, this study confirms the protective role of TYK2^P^ by reducing IL-12, IL-23, and IFN-α signalling.

Another interesting experimental study showed that the suppressor of T cell receptor signalling 1 (STS-1) tyrosine phosphatase was overexpressed in lupus B cells and indirectly activated the JAK–STAT pathway to induce IFN-α-mediated autophagy. Specifically, the activation of the JAK–STAT pathway was mediated by IFN-α-induced phosphorylation of TYK2 as a result of decreased phosphorylation of the E3 ubiquitin protein ligase c-cbl by STS-1. Thus, TYK2 seems to play an important role in the increased autophagy activity of SLE B cells (33).

TYK2 has also been implicated in neuropsychiatric SLE (NPSLE). Recent studies in the field demonstrated that microglia cells are activated towards an inflammatory state involving the IL12/IL23p40 pathway in diffuse NPSLE (34). Specifically, IL-12/23p40 signalling mediated neuropsychiatric changes in systemic lupus *via* TYK2 phosphorylation in the median prefrontal cortex (35). These findings have important clinical implications that point to TYK2 inhibitors as a potential drug for diffuse NPSLE.

### 3.2. Human studies in SLE

#### 3.2.1. Janus kinase 1–3

Specific mutations or polymorphisms in JAK or STAT genes have been associated with various disorders, including immunodeficiencies, haematological conditions, and autoimmune diseases (3). This is an expected association since multiple cytokines and other mediators act through this pathway. Regarding rheumatic diseases, JAK2 polymorphisms are common in patients with Beçhet’s disease, while STAT3 and STAT6 polymorphisms have been associated with RA (36, 37). Notably, STAT4 polymorphisms have been linked to SLE susceptibility, and particularly with severe renal insufficiency in LN (38, 39).

*Ex vivo* studies in human SLE have shown that the JAK–STAT pathway upregulates the expression of IFN-regulated factor (IRF)-related genes in lupus T cells (40). Another study demonstrated that plasma cell niche cytokines, including IL-6, IL21, CXCL2, BAFF, and APRIL, enhance the production of autoantibodies and IgG in SLE-derived antibody-secreting cells in a STAT3-dependent manner (41). Further *ex vivo* studies are required to determine the role of the JAK–STAT pathway in human SLE.

#### 3.2.2. Tyrosine kinase 2

TYK2 polymorphisms have been implicated as susceptibility factors for SLE, particularly cutaneous SLE manifestations (42, 43). More specifically, the TYK2 s2304256 polymorphism has been associated with SLE in individuals of European ancestry (44) while the rs280519 polymorphism has been linked to SLE in patients of both Asian and Caucasian ancestry. Other TYK2 single-nucleotide polymorphisms (SNPs) and gene–gene interactions between TYK2 and IRF5 have been detected in Han Chinese SLE patients (45). However, the association between TYK2 polymorphisms and susceptibility to SLE has not been confirmed in all populations (46, 47). Additionally, the SNP rs34536443 confers a protective role against autoimmunity (31).

Mechanistically, cutaneous lupus erythematosus (CLE) is initiated by keratinocytes producing IFN-κ, which further promotes IFN responses and increases the keratinocyte sensitivity to UV light. This mechanism is dependent on the expression of TYK2 (48). Authors have speculated that TYK2 mediates CLE *via* the regulation of autocrine IFN-κ activity, but further studies are required to prove this hypothesis. Another study showed that the STAT4 risk allele rs7574865[T] was associated with an elevated expression of IL-12 inducing IFN-γ production in lupus T cells. This mechanism was dependent on TYK2, as its inhibition reversed IFN-γ-induced activation of immune cells (49).

## 4. JAK inhibitors in human SLE

### 4.1. Janus kinase1/3 inhibition: tofacitinib

Numerous translational studies conducted on murine models of lupus have demonstrated the robust therapeutic efficacy of tofacitinib, particularly for various manifestations such as cardiovascular disease. These findings led to the first phase 1 double-blinded safety trial, which yielded encouraging results (14). In this study, thirty patients diagnosed with SLE were randomly assigned to receive tofacitinib 5 mg or placebo (2:1) twice daily. The primary endpoints of the trial were the safety and tolerability of the drug, while secondary outcomes focused on its clinical efficacy and mechanistic studies. The results showed that treatment with tofacitinib was associated with a decrease in high-density lipoprotein cholesterol levels, cholesterol efflux capacity, and lecithin, as well as improvements in endothelium-dependent vasorelaxation and arterial stiffness. Furthermore, the drug decreased the type I IFN signature, low-density granulocytes, and circulating neutrophil extracellular traps (NETs), which were considered as mechanistic outcomes. It is noteworthy that greater drug potency was observed in SLE patients with the STAT4 risk allele.

A case series comprising ten SLE patients with active skin and/or musculoskeletal disease demonstrated adequate efficacy of tofacitinib at a dose of 5 mg twice daily throughout a follow-up period of up to 12 months (50). Seven out of ten patients achieved clinical remission with a rapid response of lupus arthritis and a partial improvement of skin rashes. However, there was no significant change in serological markers. Nonetheless, it is essential to note that this study involved a limited number of patients and had a short observation period.

### 4.2. Janus kinase1/2 inhibition: baricitinib

Baricitinib is an oral, selective inhibitor of JAK1/JAK2 that has been approved for the treatment of immune-mediated diseases, including COVID-19 (51). The first phase 2 randomised controlled trial (RCT) of baricitinib in SLE evaluated baricitinib in combination with standard of care and demonstrated efficacy in minimising lupus disease activity (52). The study included 314 SLE patients with active skin or joint disease, who were randomised to receive baricitinib at 2 different doses (2 mg or 4 mg) or placebo. The primary endpoint was the resolution of cutaneous or joint manifestations at week 24 according to the Systemic Lupus Erythematosus Disease Activity Index 2000 (SLEDAI-2K). In this study, the 4 mg dose of baricitinib exhibited clinical improvement with respect to lupus rashes and arthritis, with no new safety concerns raised during the trial. Early post-hoc analyses showed that baricitinib reduced both anti-dsDNA titres and key pro-inflammatory cytokines that are upregulated in SLE, suggesting a multitargeted mechanism of action (53, 54).

Based on the encouraging results of the phase 2 RTC of baricitinib, two phase 3 RTCs were designed to evaluate the potential efficacy of baricitinib in SLE, named SLE-BRAVE 1 and SLE-BRAVE 2, involving 760 and 775 SLE patients, respectively (15, 16). Participants were randomly assigned 1:1:1 to receive 2 mg or 4 mg of baricitinib or placebo once daily for 52 weeks. The primary endpoint was SLE Responder Index 4 (SRI-4) response at week 52 in the 4 mg baricitinib group compared with placebo. SLE-BRAVE 1 met its primary endpoint for the baricitinib 4 mg arm. However, the findings were not replicated in SLE-BRAVE 2. No safety concerns were reported in either one of the trials. Despite the mixed results from the phase 3 trials of baricitinib, JAK inhibitors still have merit as potential drugs for treating SLE, and further studies are needed to fully evaluate their efficacy and safety.

### 4.3. Selective TYK2 inhibition: deucravacitinib

In a phase 1 clinical trial that involved both human samples and animal models of LN, the administration of an oral TYK2 inhibitor (BMS-986165) was shown to block multiple autoimmune pathways in human Th1, Th17, B cells, and myeloid cells, indicating a therapeutic potential (55). Increasing evidence for the efficacy of JAK inhibitors in other immune-mediated diseases and murine studies of lupus led to the first RCT of deucravacitinib, an oral, selective, allosteric TYK2 inhibitor, in SLE (17). This study was a phase 2, dose-determination trial that included 363 patients with active SLE. The primary endpoint was SRI-4 response at week 32, while secondary outcomes included attainment of Lupus Low Disease Activity State (LLDAS), British Isles Lupus Assessment Group-based Composite Lupus Assessment (BICLA) response, Cutaneous Lupus Erythematosus Disease Area and Severity Index 50 (CLASI-50), improvements in arthritis (swollen and tender joints), and SRI-4 response at week 48. Treatment with deucravacitinib was associated with higher SRI-4 response frequencies compared with placebo. Moreover, deucravacitinib was linked to better outcomes in terms of LLDAS, BICLA, CLASI-50, and joint counts. The optimal dose of deucravacitinib was found to be 3 mg twice daily, and the safety profile of the drug was acceptable. The potential efficacy of deucravacitinib will be investigated in larger phase 3 RTCs (NCT05620407, NCT05617677).

Design of RCTs and key outcomes are summarised in Table 2.

**Table 2:** Randomized clinical trials of Janus kinase (JAK) inhibitors in systemic lupus erythematosus.

Drug (dose)	Inhibition	Phase	Number of patients	Primary endpoints	Outcome	Safety concerns*^#^*	Reference
Tofacitinib (5 mg twice daily)	JAK1/JAK3	1	30	Safety and tolerability	Well-tolerated with no safety issues	No*, ^&^	Hasni et al., 2021 (14)
Baricitinib (2 mg or 4 mg once daily)	JAK1/JAK2	2	314	Resolution of cutaneous or joint manifestations at week 24	Significant clinical improvement in lupus rashes and arthritis	No^&^	Wallace et al., 2018 (52)
Baricitinib (2 mg or 4 mg once daily)	JAK1/JAK2	3	760	SLE Responder Index 4 (SRI-4) response at week 52 in the 4 mg baricitinib group	Primary endpoint was met	No^&^	Morand et al., 2023 (15)
Baricitinib (2 mg or 4 mg once daily)	JAK1/JAK2	3	775	SLE Responder Index 4 (SRI-4) response at week 52 in the 4 mg baricitinib group	Primary endpoint was not met	No^&^	Petri et al., 2023 (16)
Deucravacitinib (3 mg or 6 mg twice daily)	TYK2	2	363	SLE Responder Index 4 (SRI-4) response at week 32	Higher SRI-4 response frequencies compared with placebo	No	Morand et al., 2023 (17)

# According to the initial report of each study.

* *Post-hoc* analysis of the ORAL surveillance trial demonstrated that RA patients with cardiovascular risk factors who were treated with tofacitinib had a higher likelihood of exhibiting cardiovascular events or developing cancer (56).

& Data from the Swedish Rheumatology Quality Register indicate that RA patients treated with tofacitinib or baricitinib are at increased risk of venous thromboembolism compared with those treated with other biologic disease-modifying anti-rheumatic drugs (DMARDs) (57).

### 4.4. Safety concerns

In a post-hoc analysis of the ORAL surveillance trial, it was found that RA patients with cardiovascular risk factors who were treated with tofacitinib had a higher likelihood of exhibiting a cardiovascular event, including myocardial infarction, stroke, and death due to cardiovascular disease (56). Additionally, these patients had a higher risk of developing non-melanoma skin cancer compared to RA patients treated with TNF-α inhibitors (56). This observation led to several real-world studies assessing the potential side effects of JAK inhibitors. Notably, a large population-based study involving 102,263 participants found no enhanced risk for cardiovascular events in RA patients conferred from tofacitinib treatment compared to treatment with anti-TNF agents (58). The same study revealed no association between the use of tofacitinib and the occurrence of any type of cancer (excluding non-melanoma skin cancer) (58). A meta-analysis including 29 RCTs showed no increased likelihood of either cardiovascular events or all-cause mortality in patients with autoimmune diseases treated with tofacitinib (59). However, real-world data from the national Swedish registry indicated that RA patients treated with tofacitinib or baricitinib are at increased risk for venous thromboembolism compared with those treated with other biologic disease-modifying anti-rheumatic drugs (DMARDs) (57). While JAK inhibitors may hold promise as potential therapies for SLE, it is essential to approach these findings with prudence owing to the heightened propensity of such agents to trigger cardiovascular events as an adverse effect, particularly in view of established knowledge that SLE patients have an increased risk of atherosclerosis and cardiovascular disease (60). To this end, it is worth mentioning that the safety of JAK inhibitors used to treat various autoimmune diseases is currently under investigation by the Pharmacovigilance Risk Assessment Committee (PRAC).

## 5. Concluding remarks

Unfortunately, the SLE community has witnessed a multitude of failures of phase 2 and 3 clinical trials (61). However, during the last two decades we have also witnessed drug development that has resulted in licenced drugs (12). Recent advancements concerning JAK inhibition have yielded promising results for the treatment of autoimmune diseases. By targeting pro-inflammatory state-mediated signalling, these small molecules offer several advantages over biologics, such as oral administration, potentially lower costs, and a broad spectrum of mode of action that includes targeting multiple cytokines simultaneously. Importantly, JAK inhibitors including tofacitinib, baricitinib, and deucravacitinib have shown potential for treating SLE. Among them, the inhibition of TYK2 emerges as a promising therapeutic strategy due to its interference with key cytokines underlying lupus pathogenesis. We are cautiously optimistic about the results of the phase 3 trials of deucravacitinib for this challenging disease.

## Author contributions

All authors listed have made a substantial, direct, and intellectual contribution to the work and approved it for publication.

## Funding

IP is supported by grants from the Swedish Rheumatism Association (R-969696), King Gustaf V’s 80-year Foundation (FAI-2020-0741), Swedish Society of Medicine (SLS-974449), Nyckelfonden (OLL-974804), Professor Nanna Svartz Foundation (2021-00436), Ulla and Roland Gustafsson Foundation (2021–26), Region Stockholm (FoUI-955483), and Karolinska Institutet.

## Conflict of interest

IP has received research funding and/or honoraria from Amgen, AstraZeneca, Aurinia, Elli Lilly, Gilead, GlaxoSmithKline, Janssen, Novartis, Otsuka, and Roche. The funders had no role in the design of the study, the analyses or interpretation of data, or the writing of the manuscript. DN declares that he has no conflicts of interest related to this work.

## Publisher’s note

All claims expressed in this article are solely those of the authors and do not necessarily represent those of their affiliated organizations, or those of the publisher, the editors and the reviewers. Any product that may be evaluated in this article, or claim that may be made by its manufacturer, is not guaranteed or endorsed by the publisher.

## References

[1] Tsokos GC. Systemic lupus erythematosus. N Engl J Med. (2011) 365:2110–21. doi: 10.1056/NEJMra1100359 22129255

[2] Mahieu MA Strand V Simon LS Lipsky PE Ramsey-Goldman R. A critical review of clinical trials in systemic lupus erythematosus. Lupus. (2016) 25:1122–40. doi: 10.1177/0961203316652492, PMID: 27497257 PMC4978143

[3] Banerjee S Biehl A Gadina M Hasni S Schwartz DM. JAK-STAT signaling as a target for inflammatory and autoimmune diseases: current and future prospects. Drugs. (2017) 77:521–46. doi: 10.1007/s40265-017-0701-9, PMID: 28255960 PMC7102286

[4] Tao MJ Cheng P Jin LR Zhou J Shi W Peng H. The safety and efficacy of biologic agents in treatment of systemic lupus erythematosus: a network meta-analysis. Pak J Med Sci. (2019) 35:1680–6. doi: 10.12669/pjms.35.6.771, PMID: 31777515 PMC6861478

[5] Nogueira M Puig L Torres T. JAK inhibitors for treatment of psoriasis: focus on selective TYK2 inhibitors. Drugs. (2020) 80:341–52. doi: 10.1007/s40265-020-01261-8, PMID: 32020553

[6] Thomas SJ Snowden JA Zeidler MP Danson SJ. The role of JAK/STAT signalling in the pathogenesis, prognosis and treatment of solid tumours. Br J Cancer. (2015) 113:365–71. doi: 10.1038/bjc.2015.233, PMID: 26151455 PMC4522639

[7] Alunno A Padjen I Fanouriakis A Boumpas DT. Pathogenic and therapeutic relevance of JAK/STAT signaling in systemic lupus Erythematosus: integration of distinct inflammatory pathways and the Prospect of their inhibition with an Oral agent. Cells. (2019) 8:898. doi: 10.3390/cells8080898, PMID: 31443172 PMC6721755

[8] Ohl K Tenbrock K. Inflammatory cytokines in systemic lupus erythematosus. J Biomed Biotechnol. (2011) 2011:432595:1–14. doi: 10.1155/2011/432595 22028588 PMC3196871

[9] Li B Jones LL Geiger TL. IL-6 promotes T cell proliferation and expansion under inflammatory conditions in association with low-level RORγt expression. J Immunol. (2018) 201:2934–46. doi: 10.4049/jimmunol.1800016, PMID: 30315140 PMC6324200

[10] Manolakou T Nikolopoulos D Gkikas D Filia A Samiotaki M Stamatakis G. ATR-mediated DNA damage responses underlie aberrant B cell activity in systemic lupus erythematosus. Sci Adv. (2022) 8:eabo5840. doi: 10.1126/sciadv.abo5840 36306362 PMC9616496

[11] Tanaka Y Luo Y O’Shea JJ Nakayamada S. Janus kinase-targeting therapies in rheumatology: a mechanisms-based approach. Nat Rev Rheumatol. (2022) 18:133–45. doi: 10.1038/s41584-021-00726-8, PMID: 34987201 PMC8730299

[12] Kostopoulou M Fanouriakis A Bertsias G Boumpas DT. Treatment of lupus: more options after a long wait. Ann Rheum Dis. (2022) 81:753–6. doi: 10.1136/annrheumdis-2021-221817, PMID: 35027404

[13] Lin CM Cooles FA Isaacs JD. Basic mechanisms of JAK inhibition. Mediterr J Rheumatol. (2020) 31:100–4. doi: 10.31138/mjr.31.1.100, PMID: 32676567 PMC7361186

[14] Hasni SA Gupta S Davis M Poncio E Temesgen-Oyelakin Y Carlucci PM. Phase 1 double-blind randomized safety trial of the Janus kinase inhibitor tofacitinib in systemic lupus erythematosus. Nat Commun. (2021) 12:3391. doi: 10.1038/s41467-021-23361-z, PMID: 34099646 PMC8185103

[15] Morand EF Vital EM Petri M van Vollenhoven R Wallace DJ Mosca M. Baricitinib for systemic lupus erythematosus: a double-blind, randomised, placebo-controlled, phase 3 trial (SLE-BRAVE-I). Lancet. (2023) 401:1001–10. doi: 10.1016/S0140-6736(22)02607-1, PMID: 36848918

[16] Petri M Bruce IN Dörner T Tanaka Y Morand EF Kalunian KC. Baricitinib for systemic lupus erythematosus: a double-blind, randomised, placebo-controlled, phase 3 trial (SLE-BRAVE-II). Lancet. (2023) 401:1011–9. doi: 10.1016/S0140-6736(22)02546-6, PMID: 36848919

[17] Morand E Pike M Merrill JT van Vollenhoven R Werth VP Hobar C. Deucravacitinib, a tyrosine kinase 2 inhibitor, in systemic lupus Erythematosus: a phase II, randomized, double-blind. Placebo-Controlled Trial Arthritis Rheumatol. (2023) 75:242–52. doi: 10.1002/art.42391, PMID: 36369798 PMC10100399

[18] Dong J Wang QX Zhou CY Ma XF Zhang YC. Activation of the STAT1 signalling pathway in lupus nephritis in MRL/lpr mice. Lupus. (2007) 16:101–9. doi: 10.1177/0961203306075383, PMID: 17402366

[19] Martinez-Lostao L Ordi-Ros J Balada E Segarra-Medrano A Majó-Masferrer J Labrador-Horrillo M. Activation of the signal transducer and activator of transcription-1 in diffuse proliferative lupus nephritis. Lupus. (2007) 16:483–8. doi: 10.1177/0961203307079618, PMID: 17670846

[20] Wang S Yang N Zhang L Huang B Tan H Liang Y. Jak/STAT signaling is involved in the inflammatory infiltration of the kidneys in MRL/lpr mice. Lupus. (2010) 19:1171–80. doi: 10.1177/0961203310367660, PMID: 20501525

[21] Tagoe C Putterman C. JAK2 inhibition in murine systemic lupus erythematosus. Immunotherapy. (2012) 4:369–72. doi: 10.2217/imt.12.20, PMID: 22512630

[22] Lu LD Stump KL Wallace NH Dobrzanski P Serdikoff C Gingrich DE. Depletion of autoreactive plasma cells and treatment of lupus nephritis in mice using CEP-33779, a novel, orally active, selective inhibitor of JAK2. J Immunol. (2011) 187:3840–53. doi: 10.4049/jimmunol.1101228, PMID: 21880982

[23] Furumoto Y Smith CK Blanco L Zhao W Brooks SR Thacker SG. Tofacitinib ameliorates murine lupus and its associated vascular dysfunction. Arthritis Rheumatol. (2017) 69:148–60. doi: 10.1002/art.39818, PMID: 27429362 PMC5195893

[24] Ripoll È de Ramon L Draibe Bordignon J Merino A Bolaños N Goma M. JAK3-STAT pathway blocking benefits in experimental lupus nephritis. Arthritis Res Ther. (2016) 18:134. doi: 10.1186/s13075-016-1034-x, PMID: 27278657 PMC4898357

[25] Ikeda K Hayakawa K Fujishiro M Kawasaki M Hirai T Tsushima H. JAK inhibitor has the amelioration effect in lupus-prone mice: the involvement of IFN signature gene downregulation. BMC Immunol. (2017) 18:41. doi: 10.1186/s12865-017-0225-9, PMID: 28830352 PMC5568047

[26] Ding C Chen X Dascani P Hu X Bolli R Zhang HG. STAT3 signaling in B cells is critical for germinal center maintenance and contributes to the pathogenesis of murine models of lupus. J Immunol. (2016) 196:4477–86. doi: 10.4049/jimmunol.1502043, PMID: 27183592 PMC4875824

[27] Wu T Ye Y Min SY Zhu J Khobahy E Zhou J. Prevention of murine lupus nephritis by targeting multiple signaling axes and oxidative stress using a synthetic triterpenoid. Arthritis Rheumatol. (2014) 66:3129–39. doi: 10.1002/art.38782, PMID: 25047252 PMC4840107

[28] Edwards LJ Mizui M Kyttaris V. Signal transducer and activator of transcription (STAT) 3 inhibition delays the onset of lupus nephritis in MRL/lpr mice. Clin Immunol. (2015) 158:221–30. doi: 10.1016/j.clim.2015.04.004, PMID: 25869298 PMC4465043

[29] Georgakis S Gkirtzimanaki K Papadaki G Gakiopoulou H Drakos E Eloranta ML. NETs decorated with bioactive IL-33 infiltrate inflamed tissues and induce IFN-α production in patients with SLE. JCI Insight. (2021) 6:e147671. doi: 10.1172/jci.insight.147671 34554930 PMC8663547

[30] Wenzel J van Holt N Maier J Vonnahme M Bieber T Wolf D. JAK1/2 inhibitor Ruxolitinib controls a case of chilblain lupus Erythematosus. J Invest Dermatol. (2016) 136:1281–3. doi: 10.1016/j.jid.2016.02.015, PMID: 26916391

[31] Dendrou CA Cortes A Shipman L Evans HG Attfield KE Jostins L. Resolving TYK2 locus genotype-to-phenotype differences in autoimmunity. Sci Transl Med. (2016) 8:363ra149. doi: 10.1126/scitranslmed.aag1974 PMC5737835 27807284

[32] Gorman JA Hundhausen C Kinsman M Arkatkar T Allenspach EJ Clough C. The TYK2-P1104A autoimmune protective variant limits coordinate signals required to generate specialized T cell subsets. Front Immunol. (2019) 10:44. doi: 10.3389/fimmu.2019.00044, PMID: 30740104 PMC6355696

[33] Dong G You M Fan H Ding L Sun L Hou Y. STS-1 promotes IFN-α induced autophagy by activating the JAK1-STAT1 signaling pathway in B cells. Eur J Immunol. (2015) 45:2377–88. doi: 10.1002/eji.201445349, PMID: 25959715

[34] Nikolopoulos D Manolakou T Polissidis A Filia A Bertsias G Koutmani Y. Microglia activation in the presence of intact blood-brain barrier and disruption of hippocampal neurogenesis via IL-6 and IL-18 mediate early diffuse neuropsychiatric lupus. Ann Rheum Dis. (2023) 82:646–57. doi: 10.1136/ard-2022-223506, PMID: 36898766 PMC10176423

[35] Abe N Tarumi M Fujieda Y Takahashi N Karino K Uchida M. Pathogenic neuropsychiatric effect of stress-induced microglial interleukin 12/23 axis in systemic lupus erythematosus. Ann Rheum Dis. (2022) 81:1564–75. doi: 10.1136/ard-2022-222566, PMID: 35817472

[36] Duetsch G Illig T Loesgen S Rohde K Klopp N Herbon N. STAT6 as an asthma candidate gene: polymorphism-screening, association and haplotype analysis in a Caucasian sib-pair study. Hum Mol Genet. (2002) 11:613–21. doi: 10.1093/hmg/11.6.613, PMID: 11912176

[37] Schwartz DM Bonelli M Gadina M O’Shea JJ. Type I/II cytokines, JAKs, and new strategies for treating autoimmune diseases. Nat Rev Rheumatol. (2016) 12:25–36. doi: 10.1038/nrrheum.2015.167, PMID: 26633291 PMC4688091

[38] Bolin K Sandling JK Zickert A Jönsen A Sjöwall C Svenungsson E. Association of STAT4 polymorphism with severe renal insufficiency in lupus nephritis. PLoS One. (2013) 8:e84450. doi: 10.1371/journal.pone.0084450, PMID: 24386384 PMC3873995

[39] Remmers EF Plenge RM Lee AT Graham RR Hom G Behrens TW. STAT4 and the risk of rheumatoid arthritis and systemic lupus erythematosus. N Engl J Med. (2007) 357:977–86. doi: 10.1056/NEJMoa073003, PMID: 17804842 PMC2630215

[40] Kawasaki M Fujishiro M Yamaguchi A Nozawa K Kaneko H Takasaki Y. Possible role of the JAK/STAT pathways in the regulation of T cell-interferon related genes in systemic lupus erythematosus. Lupus. (2011) 20:1231–9. doi: 10.1177/0961203311409963, PMID: 21980035

[41] de la Varga MR Rodríguez-Bayona B Añez GA Medina Varo F Pérez Venegas JJ Brieva JA. Clinical relevance of circulating anti-ENA and anti-dsDNA secreting cells from SLE patients and their dependence on STAT-3 activation. Eur J Immunol. (2017) 47:1211–9. doi: 10.1002/eji.201646872 28463395

[42] Cunninghame Graham DS Morris DL Bhangale TR Criswell LA Syvänen AC Rönnblom L. Association of NCF2, IKZF1, IRF8, IFIH1, and TYK2 with systemic lupus erythematosus. PLoS Genet. (2011) 7:e1002341. doi: 10.1371/journal.pgen.1002341, PMID: 22046141 PMC3203198

[43] Järvinen TM Hellquist A Koskenmies S Einarsdottir E Koskinen LL Jeskanen L. Tyrosine kinase 2 and interferon regulatory factor 5 polymorphisms are associated with discoid and subacute cutaneous lupus erythematosus. Exp Dermatol. (2010) 19:123–31. doi: 10.1111/j.1600-0625.2009.00982.x, PMID: 19758313

[44] Lee YH Choi SJ Ji JD Song GG. Associations between PXK and TYK2 polymorphisms and systemic lupus erythematosus: a meta-analysis. Inflamm Res. (2012) 61:949–54. doi: 10.1007/s00011-012-0486-y, PMID: 22592861

[45] Tang L Wan P Wang Y Pan J Chen B. Genetic association and interaction between the IRF5 and TYK2 genes and systemic lupus erythematosus in the Han Chinese population. Inflamm Res. (2015) 64:817–24. doi: 10.1007/s00011-015-0865-2, PMID: 26294277

[46] Kyogoku C Morinobu A Nishimura K Sugiyama D Hashimoto H Tokano Y. Lack of association between tyrosine kinase 2 (TYK2) gene polymorphisms and susceptibility to SLE in a Japanese population. Mod Rheumatol. (2009) 19:401–6. doi: 10.3109/s10165-009-0173-1, PMID: 19440814

[47] Li P Chang YK Shek KW Lau YL. Lack of association of TYK2 gene polymorphisms in Chinese patients with systemic lupus erythematosus. J Rheumatol. (2011) 38. Canada:177–8. doi: 10.3899/jrheum.100424 21196586

[48] Sarkar MK Hile GA Tsoi LC Xing X Liu J Liang Y. Photosensitivity and type I IFN responses in cutaneous lupus are driven by epidermal-derived interferon kappa. Ann Rheum Dis. (2018) 77:1653–64. doi: 10.1136/annrheumdis-2018-213197, PMID: 30021804 PMC6185784

[49] Hagberg N Joelsson M Leonard D Reid S Eloranta ML Mo J. The STAT4 SLE risk allele rs7574865[T] is associated with increased IL-12-induced IFN-γ production in T cells from patients with SLE. Ann Rheum Dis. (2018) 77:1070–7. doi: 10.1136/annrheumdis-2017-212794, PMID: 29475858 PMC6029643

[50] You H Zhang G Wang Q Zhang S Zhao J Tian X. Successful treatment of arthritis and rash with tofacitinib in systemic lupus erythematosus: the experience from a single Centre. Ann Rheum Dis. (2019) 78. England:1441–3. doi: 10.1136/annrheumdis-2019-215455 31005902

[51] Bieber T Feist E Irvine AD Harigai M Haladyj E Ball S. A review of safety outcomes from clinical trials of Baricitinib in rheumatology, dermatology and COVID-19. Adv Ther. (2022) 39:4910–60. doi: 10.1007/s12325-022-02281-4, PMID: 36063279 PMC9443639

[52] Wallace DJ Furie RA Tanaka Y Kalunian KC Mosca M Petri MA. Baricitinib for systemic lupus erythematosus: a double-blind, randomised, placebo-controlled, phase 2 trial. Lancet. (2018) 392:222–31. doi: 10.1016/S0140-6736(18)31363-1, PMID: 30043749

[53] Dörner T van Vollenhoven RF Doria A Jia B Ross Terres JA Silk ME. Baricitinib decreases anti-dsDNA in patients with systemic lupus erythematosus: results from a phase II double-blind, randomized, placebo-controlled trial. Arthritis Res Ther. (2022) 24:112. doi: 10.1186/s13075-022-02794-x, PMID: 35578304 PMC9109322

[54] Dörner T Tanaka Y Dow ER Koch AE Silk M Ross Terres JA. Mechanism of action of baricitinib and identification of biomarkers and key immune pathways in patients with active systemic lupus erythematosus. Ann Rheum Dis. (2022) 81:1267–72. doi: 10.1136/annrheumdis-2022-222335, PMID: 35609978 PMC9380497

[55] Burke JR Cheng L Gillooly KM Strnad J Zupa-Fernandez A Catlett IM. Autoimmune pathways in mice and humans are blocked by pharmacological stabilization of the TYK2 pseudokinase domain. Sci Transl Med. (2019) 11:eaaw1736. doi: 10.1126/scitranslmed.aaw1736, PMID: 31341059

[56] Ytterberg SR Bhatt DL Mikuls TR Koch GG Fleischmann R Rivas JL. Cardiovascular and Cancer risk with Tofacitinib in rheumatoid arthritis. N Engl J Med. (2022) 386:316–26. doi: 10.1056/NEJMoa2109927, PMID: 35081280

[57] Molander V Bower H Frisell T Delcoigne B Di Giuseppe D Askling J. Venous thromboembolism with JAK inhibitors and other immune-modulatory drugs: a Swedish comparative safety study among patients with rheumatoid arthritis. Ann Rheum Dis. (2023) 82:189–97. doi: 10.1136/ard-2022-223050, PMID: 36150749 PMC9887398

[58] Khosrow-Khavar F Kim SC Lee H Lee SB Desai RJ. Tofacitinib and risk of cardiovascular outcomes: results from the safety of TofAcitinib in routine care patients with rheumatoid arthritis (STAR-RA) study. Ann Rheum Dis. (2022) 81:798–804. doi: 10.1136/annrheumdis-2021-221915, PMID: 35027405 PMC9117457

[59] Xie W Xiao S Huang Y Sun X Zhang Z. Effect of tofacitinib on cardiovascular events and all-cause mortality in patients with immune-mediated inflammatory diseases: a systematic review and meta-analysis of randomized controlled trials. Ther Adv Musculoskelet Dis. (2019) 11:1759720X1989549. doi: 10.1177/1759720X19895492 PMC6918042 31897092

[60] Kostopoulou M Nikolopoulos D Parodis I Bertsias G. Cardiovascular disease in systemic lupus Erythematosus: recent data on epidemiology, risk factors and prevention. Curr Vasc Pharmacol. (2020) 18:549–65. doi: 10.2174/1570161118666191227101636, PMID: 31880245

[61] Nikolopoulos D Fotis L Gioti O Fanouriakis A. Tailored treatment strategies and future directions in systemic lupus erythematosus. Rheumatol Int. (2022) 42:1307–19. doi: 10.1007/s00296-022-05133-0, PMID: 35449237

